# Research progress in the development of natural-product-based mucosal vaccine adjuvants

**DOI:** 10.3389/fimmu.2023.1152855

**Published:** 2023-04-05

**Authors:** Yingying Gao, Ying Guo

**Affiliations:** Department of Clinical Laboratory, Affiliated Banan Hospital of Chongqing Medical University, Chongqing, China

**Keywords:** mucosal vaccines, adjuvants, polysaccharides, saponins, natural-product-based

## Abstract

Mucosal vaccines have great potential and advantages in preventing infection caused by multiple pathogens. In developing mucosal vaccines, the biggest challenge comes from finding safe and effective adjuvants and drug delivery systems. Great progress has been made in the generation of mucosal adjuvants using detoxified bacterial toxin derivatives, pathogen-related molecules, cytokines, and various vaccine delivery systems. However, many problems, relating to the safety and efficacy of mucosal vaccine adjuvants, remain. Certain natural substances can boost the immune response and thus could be used as adjuvants in vaccination. These natural-product-based immune adjuvants have certain advantages over conventional adjuvants, such as low toxicity, high stability, and low cost of production. In this review, we summarize the latest natural-product-based immune adjuvants, and discuss their properties and clinical applications.

## 1 Introduction

The term mucosal immune system (MIS) refers to immune cells that are widely distributed among lymphatic tissues such as those of the respiratory, gastrointestinal, and urogenital tracts, and some exocrine glands. The MIS performs the majority of localized immune functions (1). In adults, the mucosal surface area (which comprises the gastrointestinal, respiratory, and urogenital tracts) is larger than 400 m2, 200 times larger than the skin surface area (2). The MIS implicates up to 80% of all immune cells and > 50% of the lymphoid tissues (3). The epithelial cells on the mucosal surface are closely aligned with each other, forming a natural barrier. Together with the skin, the mucosal tissues ensure that pathogenic microorganisms or other foreign bodies are destroyed before they enter the underlying tissues. Moreover, mucosal epithelial cells and the associated secretory glands (e.g., the salivary glands) can secrete various mucins, bactericidal proteins, and other bactericidal substances to assist in the elimination of pathogens (4, 5). Mucosal immunization induces the earlier appearance of mucosal local antibodies, which are present at higher titers and persist for longer than serum antibodies (6). Therefore, mucosal immunity constitutes the first line of defense against infection and is incredibly important for the body’s immune function (7).

The MIS is different from the traditional immune system in both structure and function. Compared with traditional injectable vaccines, mucosal vaccines have the advantage of inducing polymorphic immune responses, which not only implicate the mucosal immune response, but also induce systemic immunity. However, a major obstacle to mucosal vaccine development is that antigens applied to the mucosa usually elicit a relatively weak immune response (8). Thus, a robust mucosal adjuvant and/or delivery system is required to produce a strong immune response.

Currently, human vaccine adjuvants on the market include aluminum hydroxide, MF59, AS01, virions, AS03, AS04, and CpG 1018 (9–11). Aluminum hydroxide adjuvants are used in more than 80% of human vaccines. Although it has been used for over 80 years, aluminum hydroxide mainly promotes humoral immunity (induced by T helper [Th]2 cells) and has little effect on cellular immunity (induce by Th1 cells), which limits its use in vaccines targeting cellular immunity (12). In addition, most reports show that adjuvants such as aluminum hydroxide can cause headache, fever, inflammation, and other adverse reactions. Therefore, the development of effective vaccine adjuvants is very important for improving the immunogenicity and safety of vaccines. Adjuvants can be synthesized artificially or obtained directly from products found in nature. Abundant natural resources are a treasure trove of adjuvants available for screening. In recent years, many effective components obtained from nature, such as polysaccharides and saponins, have been reported to promote specific and non-specific immunity in the body. These natural products have shown promising potential as vaccine adjuvant candidates because of their biocompatibility, independent, safety (i.e., low toxicity and few side effects), and biodegradability (13–15). For these reasons, natural-product-based adjuvants are being viewed as ideal immunomodulators in vaccine adjuvant research. In this paper, we review the characteristics and applications of some recently discovered natural-product-based mucosal immune adjuvants to inform future mucosal vaccine research.

## 2 Mucosal vaccines

Our increased understanding of the MIS has led to the development of a number of mucosal vaccines, some of which have played an important role in disease prevention. These vaccines can be divided into oral and nasal types, according to the route of administration and the type of immune pathway being targeted; these can be further subdivided into live attenuated or viral vector types, according to their active components.

Oral mucosal vaccination involves immunization with attenuated pathogens or their active components *via* the digestive tract. This confers immune protection at the mucosal surface of the digestive system, which mainly targets diseases spread by fecal-oral transmission. By contrast, the main objective of nasal mucosal vaccination is the prevention of infection with respiratory pathogens. In addition, transvaginal or rectal mucosal immunization is also available; however, the technology is still immature and its efficacy remains to be tested (16).

Attenuated live vaccine refers to a type of vaccine that maintains antigenicity while reducing the virulence of the pathogen after treatment. Once inoculated into the body, this type of pathogen will not cause disease. Instead, it triggers the body’s immune response, stimulates the production of specific immune cells, and plays a role in maintaining long-term protection. Viral vector vaccines are mainly used to transmit antigen genes to human cells by infecting them with replication-defective viruses as vectors. This leads to the expression of external antigens and stimulates the body to produce an immune response (17).

**Figure 1:**
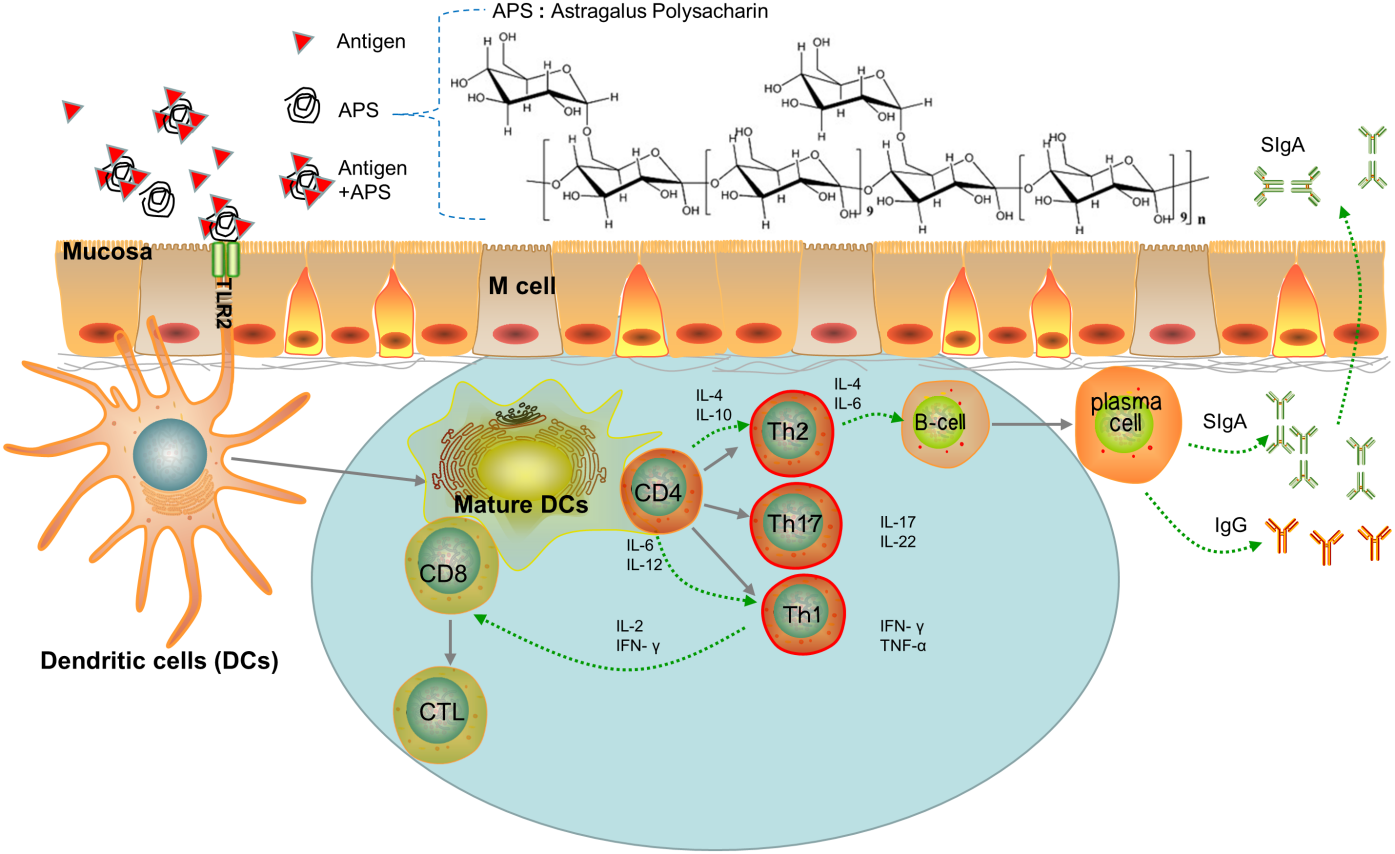
Paradigm of APS as Mucosal Immune Adjuvant. (i) Adjuvants are recognized by the TLR2 ligand of DCs and promote naive T differentiation. (ii) Th cells promote B cell differentiation into plasmacells which secrete IgA or IgG. (iii) DCs presents antigens that promote the transformation of CD8 cells into cytotoxic T lymphocytes (CTL).

A major advantage of mucosal immunization is that there is no need for injection. This is especially important in developing countries, where the underdeveloped medical conditions mean that mucosal vaccines are more likely to be popularized. Mucosal immunity not only induces the production of antigen-specific immunoglobulin (Ig)G in the serum, but also generates a secretory IgA antibody response (**Figure 1**). The IgA molecules form a protective net on the surface of mucosal cells (18), which provides better and more direct protection against infection *via* the mucosal route. However, faced with adversities such as low-level or short-lived mucosal immune responses, researchers are further exploring the mechanism of the mucosal-vaccine-induced immune response with the aim of developing safer and more effective mucosal vaccine adjuvants and vectors. The hope is that these advances will increase the potency and application of mucosal vaccines.

## 3 Mucosal vaccine adjuvants

Under normal circumstances, muco-associated lymphoid tissues can produce an immune response to harmful, foreign antigenic substances that attempt to enter the body *via* the mucous membranes. Mucosal immunization can effectively induce local and systemic immune responses. However, most of the existing mucosal antigens are weakly immunogenic when they are administered *via* mucous membranes, especially *via* the oral route. This means that a robust immune response can only be achieved with a long course of repeated vaccinations. The mucosal-vaccine-induced immune response is short-lived and can easily cause immune tolerance, which has hindered further research and development of mucous membrane vaccines. To date, with the exception of the oral polio vaccine, few mucosal vaccines have been used effectively in humans. Cholera toxins and Escherichia-coli-derived heat-resistant toxins are good mucosal adjuvants; however, both can cause severe diarrhea (19) and are not suitable for human use because of their potential damaging effects on the central nervous system. Therefore, the search for efficient, safe, and suitable mucosal adjuvant is a pressing issue in mucosal vaccine research.

Generally, the potency of an adjuvant depends on the cell receptor associated with it, its structure and physicochemical properties, and the type of antigen (20). Mucosal adjuvants, including proteotoxins, recombinant protein subunits, and nucleic acids, can be divided into two categories: immune stimulants and immune delivery systems. Immunostimulating adjuvants (e.g., LT, CT, TLR ligands, and cytokines) can directly activate natural immunity, while the immune delivery systems (e.g., virus-like particles, liposomes, and protoplasts) can protect the antigen and mucosal surface targeted M cells under harsh conditions (21).

In recent years, many active natural-product-based ingredients, such as polysaccharides and glycosides, have been shown to improve the recognition of antigens by immune cells (e.g., macrophages, natural killer [NK] cells, cytotoxic cells, and lymphokine-activated killer cells), promote the secretion of cytokines, activate B and T lymphocytes, activate complement, and regulate a variety of biological reactions (**Table 1**),. In addition, most natural products exert their biological effects mainly through mucosal contact, which is associated with minimal side effects and low levels of tolerance induction. These natural-product-based compounds can therefore be used to develop new human mucosal vaccine adjuvants, which meet the development requirements of new vaccines and have broad application prospects.

**Table 1:** List of some natural-product-based mucosal vaccine adjuvants and their lmmunomodulatory effect.

Mucosal Vaccine Adjuvants		Subjects	Immunomodulatory Activity	References
Polysaccharides	Chitosan	mouse	Increasing the release of interleukin (IL)-1β, IL-6, and tumor necrosis factor (TNF)-α, promoting the proliferation and differentiation of CD4+ and CD8+ T cells, stimulating humoral and cellular immunity, and inducing natural and acquired immune responses. Facilitating the recognition of antigens by mucosal APCs and leading to a series of immune responses. Protecting the antigen from degradation, and significant increasing in IgG and IgA titers.	(22–24)
		humans	Inducing IgA secretion in mucosal tissue.	(25)
	Acetylated dextran	mouse	Stronging IgG, IgG1, and IgG2a responses, enhancing cellular uptake and the activation of certain intracellular pathways, producing a more balanced Th1/Th2 response.	(26)
	Fungal polysaccharide	mouse	Reducing the OVA-induced Th2 immune response and enhancing the Th1 immune response.	(27)
	β-glucan	mouse	Increasing the number of intestinal intra-epithelial lymphocytes and IFN-γ levels, causing a rapid T cell response in the mucosa.	(28)
	Mannose	mouse	Targeting DCs and significantly inducing DCs maturation. Decreasing the proportion of regulatory T cells, while increasing the number of IFN-γ-positive cells and serum IgG levels.	(29)
	QuilA and QS-21	mouse	Activating the Th1 immune response, promoting the expansion of cytotoxic T lymphocytes to eradicate endogenous antigens, and inducing the maturation of DCs.	(30)
Saponins	Ginsenosides Rg1/R3/GS-R	mouse	Stimulating the production of IgG, IFN-γ, and IL-4, activating DCs. Inducting the production of IFN-γand IL-2. Enhancing the Th1/Th2 immune response.	(31–33)
	Albizia saponins	mouse	Improving the antigen-specific cellular and humoral immune responses by inducing cytokine and chemokine production, triggering Th1/Th2 responses.	(34)
	Platycodin	mouse	Improving the absorption of OVA from mouse bone-marrow-derived DCs, reducing the toxicity of pantocoside, and inducing a balanced Th1/Th2-mediated humoral immune response.	(35)

## 4 Natural-product-based mucosal vaccine adjuvants

### 4.1 Polysaccharides

Polysaccharides are glycans produced by plant cell metabolism. These compounds are ubiquitous in nature and exhibit a high degree of polymerization (> 10 units). Natural polysaccharides (e.g., including starch, cellulose, polysaccharide, and pectin) have intrinsic functions such as immune regulation, anti-tumor properties, hypoglycemic and lipid-lowering potential, good biocompatibility and biodegradability, low toxicity, and high safety.

In recent years, many studies have confirmed that plant polysaccharides can be used as immune adjuvants. Polysaccharide adjuvants can activate macrophages, T lymphocytes, B lymphocytes, and NK cells and promote the secretion of immune-related molecules (such as cytokines, antibodies, and complement). They have also been show to improve humoral, cellular, and mucosal immunity to achieve tumor eradication (36). Polysaccharide adjuvants are mainly based on chitosan, glucan, and mannose.

#### 4.1.1 Chitosan

Chitosan is naturally formed by chitino-N-acetylation. It is the only cationic polysaccharide (37), whose properties as an immune adjuvant were first described by Nishimura et al. (38) in 1984. Chitosan alone cannot induce a T-cell-related immune response. However, chitosan and its derivatives become more effective when combined with antigens. Chitosan granules stimulate the immune response in various ways, such as by increasing the release of interleukin (IL)-1β, IL-6, and tumor necrosis factor (TNF)-α, promoting the proliferation and differentiation of CD4+ and CD8+ T cells, stimulating humoral and cellular immunity, and inducing natural and acquired immune responses (22). Chitosan is released in a slow and controlled manner, which prolongs the action time of the associated antigen or drug in the gastrointestinal tract. In recent years, chitosan has shown application potential as a mucosal vaccine adjuvant. Due to its unique cationic properties, chitosan adheres well to the mucosal surface, which not only prolongs the retention time of antigens but also opens tight junctions between mucosal epithelial cells so that antigens can be more readily accessed by mucosal antigen presenting cells (23). Almasian et al. (24) immunized BALB/c mice with an oral mucosal vaccine, which was generated using chitosan nanoparticles containing the E. coli Stx2B protein. The results confirmed that chitosan nanoparticles protected the antigen from degradation and controlled antigen release in the gastrointestinal tract, as evidenced by the significant increase in IgG and IgA titers in immunized vs. control mice. A phase I clinical trial of a powder spray vaccine (NV-VLP) made from chitosan (ChiSysTM), monophosphoyl lipid (MPL), and norovirus VLP antigen has shown that this vaccine was well tolerated in humans (with no serious adverse effects) and induced IgA secretion in intestinal mucosal tissue (the NV entry site) (25).

#### 4.1.2 Glucans

Glucans can enhance the immune response to the vaccine. Two types of glucans exist: α-glucans and β-glucans, whereby α-glucans comprise mainly dextran (a bacterial polysaccharide), while β-glucans comprise polysaccharides from organisms such as algae, yeast, and fungi. Glucans can enhance the immune response to a vaccine and increase serum antibody titers and the levels of cytokines such as IL-2 and interferon (IFN)-γ (39). In addition, Mirza et al. (40) found that different compounds (including protein antigens) could be loaded into the internal cavity of glucan granules and transported to macrophages and DCs. Thus, glucans can be used as both adjuvants and delivery systems for targeted antigen presentation. The preparation of glucan as an antigenic carrier can also induce a strong immune response. Deepe et al. (41) immunized mice with dextran particles coated with a basic extract of Histoplasma capsulatum. They found that the number of colonies in the lung and spleen decreased significantly, while the secretion of IFN-γ and IL-17 in the lung increased, indicative of Th1 and Th17 immune responses.

##### 4.1.2.1 α-glucans

Dextran is a homopolysaccharide formed from the condensation of multiple glucose molecules. Dextran sulphate has extensive proinflammatory properties and is an effective immune adjuvant used to induce a cell-mediated delayed hypersensitivity reaction (42). Its derivative, diethylaminoethyl (DEAE)-glucan, has been used as an adjuvant in veterinary practice. Chen et al. (26) found that mice immunized with acetylated dextran (Ac-DEX) particles as adjuvants and ovalbumin (OVA) had stronger IgG, IgG1, and IgG2a responses. This may have been due to enhanced cellular uptake and the activation of certain intracellular pathways. In addition, Ac-DEX microparticles altered the timing of peak immunity by modulating antigen release and produced a more balanced Th1/Th2 response compared to that induced in the aluminum adjuvant group. Such findings provide a research basis for the production of safer and more effective vaccine adjuvants.

##### 4.1.2.2 β-glucans

β-glucans are mainly derived from the cell walls of organisms such as yeast, algae, and fungi. Interactions between β-glucans and their receptors trigger intracellular signals that activate the expression of immune-related molecules and regulate immune responses. Studies have shown that fungal polysaccharide can reduce the OVA-induced Th2 immune response and enhance the Th1 immune response, indicating that it can activate both the innate and adaptive branches of immunity (27).

Studies on the immunity of glucans administered *via* the mucosal route have shown that after oral administration of β-glucan to mice, the number of intestinal intra-epithelial lymphocytes and IFN-γ levels increased, causing a rapid T cell response in the mucosa (28). In the early stage of pathogen exposure, the mucosa rapidly generates an immune response to prevent tissue infection. β-glucans have no apparent toxicity or side effects; however, structurally different β-glucans elicit different immune effects.

#### 4.1.3 Mannose

The function of mannose as an adjuvant depends on its binding to lectins on the surface of macrophages and DCs, then to activate complement and opsonophagocytosis. Yang et al. (29) generated lipid nanoparticles using synthetic CpG oligodeoxynucleotide (CPG-ODN) and mannose. They then loaded them with H22 liver cancer cell lysate to produce M/Cpg-Odn-H22lipo nanoparticles, which could selectively target DCs and significantly induce DCs maturation. These novel nanoparticles also decreased the proportion of regulatory T cells, while increasing the number of IFN-γ-positive cells and serum IgG levels in the spleen. Ultimately, M/Cpg-Odn-H22lipo effectively inhibited the growth of mouse liver tumors and prolonged animal survival.

In addition, scientists have researched the efficacy of immune adjuvants such as inulin, Astragalus polysaccharide, Angelica sinensis polysaccharide (ASP), Lycium barbarum polysaccharide, Radix isatidis polysaccharide, Poria polysaccharide, L. barbarum polysaccharide, and Radix achyranthes polysaccharide. Inulin, Astragalus polysaccharide, and ASP have important applications as immune adjuvants. For instance, Astragalus polysaccharide plays an immunomodulatory role by promoting the phagocytosis of reticuloendothelial system phagocytes, lymphocyte transformation, T and B cell activation, and antibody generation. Additionally, it and has been shown to enhance the immunogenicity of various antigens, including those derived from the foot-and-mouth disease virus, avian influenza virus, and hepatitis B virus (43). Moreover, as an adjuvant used in the Newcastle disease virus vaccine, Angelica polysaccharide improves the hemagglutination response and antibody titer in the later stages of immunization (44). The L. barbarum polysaccharide liposome can also promote the expression of genes encoding TLR4, MyD88, TRAF6, and NF-κB (45).

### 4.2 Saponins

Saponins are glycosides that are widely found in plants and have a variety of biological and pharmacological activities. They can activate the immune system of mammals and induce the differentiation and maturation of various immune-related cells (e.g., neutrophils, macrophages, and DCs) and cytokine production (46). Therefore, saponins have great potential as immune adjuvants. At present, triterpenoids are the main type of saponins being used as adjuvants. When used as adjuvants, different triterpenoid saponins have different pharmacological effects and mechanisms of action, according to the number of carbon rings present in their aglycones. Among these triterpenoid saponins, QuilA and its derivative QS-21, which are extracted from soap bark, are the most widely used and their effects have been verified in a large number of clinical trials (47). QuilA and QS-21 specifically activate the Th1 immune response, promote the expansion of cytotoxic T lymphocytes to eradicate endogenous antigens, and induce the maturation of DCs (30). This makes them ideal candidates for the preparation of certain vaccines or subunit vaccine adjuvants, which can be used to kill intracellular pathogens directly or in the preparation of anticancer vaccines. However, the use of QuilA and QS-21 as adjuvants has many serious drawbacks, such as their high toxicity, their ability to induce a hemolytic reaction, and their poor stability in the aqueous phase; all of these factors limit the application of such saponins as vaccine adjuvants (48). Therefore, many researchers have focused on generating saponin adjuvants from natural products other than soap bark.

Ginseng contains a lot of ginsenosides, which are the main active ingredients of ginseng. Several studies have shown that ginsenosides are effective immune adjuvants. For instance, ginsenoside Rg1 enhances the mouse immune response to hepatitis B surface antigen by (1): stimulating the production of IgG, IFN-γ, and IL-4, and activating DCs; and (2) inducing a protective cell response against lymphoma (31). Ginsenoside Rg3 has been used as an adjuvant in conventional cancer therapy, whereby it played a synergistic role by improving vaccine efficacy while reducing adverse reactions *via* the induction of IFN-γ and IL-2 production (32). Moreover, a rapeseed oil emulsion containing ginsenoside GS-R, used as an adjuvant in a foot-and-mouth disease virus vaccine, significantly enhanced the Th1/Th2 immune response (33); overall, the efficacy of this new formulation was not significantly different to that of the commercial oil adjuvant ISA206, but had the added benefit of lower viscosity.

Besides ginsenosides, other saponins can also be used as immune adjuvants. Sun et al. (34) extracted total saponin (AJSt) from the stem covering of the Ajuga reptans plant, in which AJSt75 is the most adjuvant-active component. The authors then showed that Ajst75 improved the antigen-specific cellular and humoral immune responses by inducing cytokine and chemokine production at the injection site while simultaneously triggering Th1/Th2 responses. Moreover, to enhance the immunogenicity of a subunit vaccine, Zhao et al. (35) loaded OVA and Platycodon-derived platycoside into liposomes, before screening them in a soluble microneedle array. The antigen-adjuvant delivery system greatly improved the absorption of OVA from mouse bone-marrow-derived DCs, reduced the toxicity of pantocoside, and induced a balanced Th1/Th2-mediated humoral immune response.

Besides the adjuvants mentioned, the potential application of other saponins and their derivatives as adjuvants should be explored. The properties of saponins makes them ideal candidates for the preparation of certain vaccine or subunit vaccine adjuvants, which can be used to kill intracellular pathogens directly or in the preparation of anticancer vaccines. However, many saponins elicit neurotoxicity, cytotoxicity, liver damage, hemolytic reactions, and intestinal permeability; moreover, they are unstable in the aqueous phase. These factors currently limit the application of certain saponins as vaccine adjuvants. Therefore, other natural sources of saponins should be considered.

## 5 Questions and challenges

It is crucial to develop new immune adjuvants that enhance the immunogenicity and minimize the toxicity of vaccines. Ideal adjuvants should have the following characteristics: (i) render weak antigens more immunogenic; (ii) promote humoral and/or cellular immune responses; (iii) do not cause harmful side effects; (iv) can be administered by different routes; (v) can be used alongside different types of antigens; (vi) can be used in immunosuppressed individuals; (vii) can effectively improve the quality of the immune response; (viii) are stable; and (ix) are cost-effective and easy to produce.

Recent efforts have focused on developing new adjuvants, especially those targeting mucosal immunity, from a wide range of natute. However, the following problems are associated with the development of natural-product-based immune adjuvants:

- The molecular adjuvant structure is often unclear, which hinders large-scale production. Most natural components are complex and not easy to purify, making it difficult to elucidate their mechanism of action. In adjuvant production, it is necessary to achieve a certain purity standard and meet quality requirements, without significantly increasing the cost of the vaccine.
- The quality standard is not easy to control. Most natural products contain a mixture of active compounds and the current common index can only test the content of one bioactive monomer component at a time, which leads to poor reproducibility of test results. It is difficult to develop a quality standard that can reflect the efficacy of adjuvants based on this index, as it does not conform to international standards and therefore cannot easily access the international market.
- There are many factors affecting natural product biological activity. It contains non-adjuvant active substances, which may affect drug stability and therefore efficacy. This also increases the difficulty of vaccine adjuvant screening.
- Further studies are needed on the optimal combination of natural-product-based adjuvants and vaccines. Current studies mainly focus on the separate use of natural-product-based adjuvants and vaccines, which only applies to inactivated vaccines. Whether or not natural-product-based adjuvants can be combined with live vaccines, and how best to combine them, is unclear.
- The immunomodulatory effect of natural-product-basedadjuvants shows a dose-effect relationship; generally, small doses enhance the immune response, while large doses are immunosuppressive. Thus, it is necessary to study the effective dose range of the adjuvant.

## 6 Future prospects

Mucosal vaccines are an important means of preventing and treating disease. A mucosal adjuvant acts as an enhancer of mucosal immunity to improve the eradication of pathogenic microorganisms. Natural-product-based mucosal immune adjuvants are easy to produce, have a low toxicity, and are highly stable. Despite the emergence of many new synthetic adjuvants, the natural world remains a treasure trove worth exploring. In particular, China has studied the medicinal applications of animal- and herb-derived compounds since ancient times. Researchers can draw on these ancient practices to continue the exploration of clinically useful natural compounds. We can conduct extensive screening in natural products with immune activity, trace the active parts of natural products that can be used as mucosal adjuvants with modern isolation and analysis means, then study the mechanism of action, and clarify the molecular structure and structure-activity relationship at the molecular level. Such endeavors will help to establish appropriate quality standards for adjuvant manufacture and improve the efficiency while reducing the toxicity of mucosal vaccine adjuvants. In addition, natural adjuvants can be reprocessed to improve their specificity and reduce their toxicity. Moreover, several adjuvants can be combined to enhance mucosal immunity through their synergistic effects. We envisage that the in-depth study of natural mucosal adjuvants and our increased understanding of the mucosal immune response will lead to the development of the next generation of natural mucosal vaccine adjuvants, which have better safety, stability, and efficacy profiles for use in clinical practice.

## Author contributions

All authors listed have made a substantial, direct, and intellectual contribution to the work, and approved it for publication.

## References

[1] Shao F Yu D Xia P Wang S. Dynamic regulation of innate lymphoid cells in the mucosal immune system. Cell Mol Immunol (2021) 18(6):1387–94. doi: 10.1038/s41423-021-00689-6 PMC8167116 33980994

[2] Woodrow KA Bennett KM Lo DD. Mucosal vaccine design and delivery. Annu Rev BioMed Eng (2012) 14:17–46. doi: 10.1146/annurev-bioeng-071811-150054 22524387

[3] Neudecker V Yuan X Bowser JL Eltzschig HK. Micrornas in mucosal inflammation. J Mol Med (Berl) (2017) 95(9):935–49. doi: 10.1007/s00109-017-1568-7 PMC5828032 28726085

[4] Hansson GC. Mucins and the microbiome. Annu Rev Biochem (2020) 89:769–93. doi: 10.1146/annurev-biochem-011520-105053 PMC8442341 32243763

[5] Lavelle EC Ward RW. Mucosal vaccines - fortifying the frontiers. Nat Rev Immunol (2022) 22(4):236–50. doi: 10.1038/s41577-021-00583-2 PMC8312369 34312520

[6] Villareall BB Melendro EI Ramos F Ximenez C. Local and systemic antibody response in Balb/C mice immunized with entamoeba histolytica trophozoites. Arch Med Res (1992) 23(1):69–72. 1308795

[7] Correa VA Portilho AI De Gaspari E. Vaccines, adjuvants and key factors for mucosal immune response. Immunology (2022) 167(2):124–38. doi: 10.1111/imm.13526 35751397

[8] Yuki Y Kiyono H. Mucosal vaccines: Novel advances in technology and delivery. Expert Rev Vaccines (2009) 8(8):1083–97. doi: 10.1586/erv.09.61 19627189

[9] Apostolico Jde S Lunardelli VA Coirada FC Boscardin SB Rosa DS. Adjuvants: Classification, modus operandi, and licensing. J Immunol Res (2016) 2016:1459394. doi: 10.1155/2016/1459394 27274998 PMC4870346

[10] Campbell JD. Development of the cpg adjuvant 1018: A case study. Methods Mol Biol (2017) 1494:15–27. doi: 10.1007/978-1-4939-6445-1_2 27718183

[11] O'Hagan DT Lodaya RN Lofano G. The continued advance of vaccine adjuvants - 'We can work it out'. Semin Immunol (2020) 50:101426. doi: 10.1016/j.smim.2020.101426 33257234

[12] Mbow ML De Gregorio E Valiante NM Rappuoli R. New adjuvants for human vaccines. Curr Opin Immunol (2010) 22(3):411–6. doi: 10.1016/j.coi.2010.04.004 20466528

[13] Honda-Okubo Y Saade F Petrovsky N. Advax, a polysaccharide adjuvant derived from delta inulin, provides improved influenza vaccine protection through broad-based enhancement of adaptive immune responses. Vaccine (2012) 30(36):5373–81. doi: 10.1016/j.vaccine.2012.06.021 PMC3401346 22728225

[14] Khademi F Taheri RA Yousefi Avarvand A Vaez H Momtazi-Borojeni AA Soleimanpour S. Are chitosan natural polymers suitable as Adjuvant/Delivery system for anti-tuberculosis vaccines? Microb Pathog (2018) 121:218–23. doi: 10.1016/j.micpath.2018.05.035 29800697

[15] Petrovsky N Cooper PD. Advax, a novel microcrystalline polysaccharide particle engineered from delta inulin, provides robust adjuvant potency together with tolerability and safety. Vaccine (2015) 33(44):5920–6. doi: 10.1016/j.vaccine.2015.09.030 PMC4639457 26407920

[16] Kilgore PB Sha J Hendrix EK Motin VL Chopra AK. Combinatorial viral vector-based and live attenuated vaccines without an adjuvant to generate broader immune responses to effectively combat pneumonic plague. mBio (2021) 12(6):e0322321. doi: 10.1128/mBio.03223-21 34872353 PMC8649767

[17] Alexander R Mestecky J. Neutralizing antibodies in mucosal secretions: Igg or iga? Curr HIV Res (2007) 5(6):588–93. doi: 10.2174/157016207782418452 18045115

[18] Holmgren J Bourgeois L Carlin N Clements J Gustafsson B Lundgren A. Development and preclinical evaluation of safety and immunogenicity of an oral etec vaccine containing inactivated e. coli bacteria overexpressing colonization factors Cfa/I, Cs3, Cs5 and Cs6 combined with a hybrid Lt/Ct b subunit antigen, administered alone and together with dmlt adjuvant. Vaccine (2013) 31(20):2457–64. doi: 10.1016/j.vaccine.2013.03.027 23541621

[19] Guerrini G Vivi A Gioria S Ponti J Magri D Hoeveler A. Physicochemical characterization cascade of nanoadjuvant-antigen systems for improving vaccines. Vaccines (Basel) (2021) 9(6):1–13. doi: 10.3390/vaccines9060544 PMC8224364 34064212

[20] Wang ZB Xu J. Better adjuvants for better vaccines: Progress in adjuvant delivery systems, modifications, and adjuvant-antigen codelivery. Vaccines (Basel) (2020) 8(1):1–20. doi: 10.3390/vaccines8010128 PMC7157724 32183209

[21] Didierlaurent AM Laupeze B Di Pasquale A Hergli N Collignon C Garcon N. Adjuvant system As01: Helping to overcome the challenges of modern vaccines. Expert Rev Vaccines (2017) 16(1):55–63. doi: 10.1080/14760584.2016.1213632 27448771

[22] Malik A Gupta M Gupta V Gogoi H Bhatnagar R. Novel application of trimethyl chitosan as an adjuvant in vaccine delivery. Int J Nanome (2018) 13:7959–70. doi: 10.2147/IJN.S165876 PMC6260144 30538470

[23] Yu M Yang Y Zhu C Guo S Gan Y. Advances in the transepithelial transport of nanoparticles. Drug Discov Today (2016) 21(7):1155–61. doi: 10.1016/j.drudis.2016.05.007 27196527

[24] Almasian P Amani J Arani FB Nazarian S Kazemi R Tabrizi NM. Preparation of chitosan nanoparticle containing recombinant stxb antigen of ehec and evaluation its immunogenicity in Balb/C mice. Iran J Microbiol (2018) 10(6):361–70. PMC6414748 30873263

[25] El-Kamary SS Pasetti MF Mendelman PM Frey SE Bernstein DI Treanor JJ. Adjuvanted intranasal Norwalk virus-like particle vaccine elicits antibodies and antibody-secreting cells that express homing receptors for mucosal and peripheral lymphoid tissues. J Infect Dis (2010) 202(11):1649–58. doi: 10.1086/657087 PMC3722871 20979455

[26] Chen N Johnson MM Collier MA Gallovic MD Bachelder EM Ainslie KM. Tunable degradation of acetalated dextran microparticles enables controlled vaccine adjuvant and antigen delivery to modulate adaptive immune responses. J Control Release (2018) 273:147–59. doi: 10.1016/j.jconrel.2018.01.027 PMC5835201 29407676

[27] Wu YS Chen S Wang W Lu CL Liu CF Chen SN. Oral administration of mbg to modulate immune responses and suppress ova-sensitized allergy in a murine model. Evid Based Complement Alternat Med (2014) 2014:567427. doi: 10.1155/2014/567427 24723960 PMC3960727

[28] Tsukada C Yokoyama H Miyaji C Ishimoto Y Kawamura H Abo T. Immunopotentiation of intraepithelial lymphocytes in the intestine by oral administrations of beta-glucan. Cell Immunol (2003) 221(1):1–5. doi: 10.1016/s0008-8749(03)00061-3 12742376

[29] Yang X Lai C Liu A Hou X Tang Z Mo F. Anti-tumor activity of mannose-Cpg-Oligodeoxynucleotides-Conjugated and hepatoma lysate-loaded nanoliposomes for targeting dendritic cells in vivo. J BioMed Nanotechnol (2019) 15(5):1018–32. doi: 10.1166/jbn.2019.2755 30890232

[30] Cibulski S Rivera-Patron M Suarez N Pirez M Rossi S Yendo AC. Leaf saponins of quillaja brasiliensis enhance long-term specific immune responses and promote dose-sparing effect in bvdv experimental vaccines. Vaccine (2018) 36(1):55–65. doi: 10.1016/j.vaccine.2017.11.030 29174676

[31] Huang Y Zou Y Lin L Zheng R. Ginsenoside Rg1 activates dendritic cells and acts as a vaccine adjuvant inducing protective cellular responses against lymphomas. DNA Cell Biol (2017) 36(12):1168–77. doi: 10.1089/dna.2017.3923 29058460

[32] Sun M Ye Y Xiao L Duan X Zhang Y Zhang H. Anticancer effects of ginsenoside Rg3 (Review). Int J Mol Med (2017) 39(3):507–18. doi: 10.3892/ijmm.2017.2857 28098857

[33] Zhang C Wang Y Wang M Su X Lu Y Su F. Rapeseed oil and ginseng saponins work synergistically to enhance Th1 and Th2 immune responses induced by the foot-and-Mouth disease vaccine. Clin Vaccine Immunol (2014) 21(8):1113–9. doi: 10.1128/CVI.00127-14 PMC4135922 24920601

[34] Sun H He S Shi M. Adjuvant-active fraction from albizia julibrissin saponins improves immune responses by inducing cytokine and chemokine at the site of injection. Int Immunopharmacol (2014) 22(2):346–55. doi: 10.1016/j.intimp.2014.07.021 25075718

[35] Zhao JH Zhang QB Liu B Piao XH Yan YL Hu XG. Enhanced immunization *Via* dissolving microneedle array-based delivery system incorporating subunit vaccine and saponin adjuvant. Int J Nanome (2017) 12:4763–72. doi: 10.2147/IJN.S132456 PMC5503490 28740383

[36] Sun B Yu S Zhao D Guo S Wang X Zhao K. Polysaccharides as vaccine adjuvants. Vaccine (2018) 36(35):5226–34. doi: 10.1016/j.vaccine.2018.07.040 30057282

[37] Cheung RC Ng TB Wong JH Chan WY. Chitosan: An update on potential biomedical and pharmaceutical applications. Mar Drugs (2015) 13(8):5156–86. doi: 10.3390/md13085156 PMC4557018 26287217

[38] Nishimura K Nishimura S Nishi N Saiki I Tokura S Azuma I. Immunological activity of chitin and its derivatives. Vaccine (1984) 2(1):93–9. doi: 10.1016/s0264-410x(98)90039-1 6397928

[39] Wang M Yang R Zhang L Meng X Fei C Zhang K. Sulfated glucan can improve the immune efficacy of Newcastle disease vaccine in chicken. Int J Biol Macromol (2014) 70:193–8. doi: 10.1016/j.ijbiomac.2014.05.048 24875318

[40] Mirza Z Soto ER Dikengil F Levitz SM Ostroff GR. Beta-glucan particles as vaccine adjuvant carriers. Methods Mol Biol (2017) 1625:143–57. doi: 10.1007/978-1-4939-7104-6_11 28584989

[41] Deepe GS Jr. Buesing WR Ostroff GR Abraham A Specht CA Huang H. Vaccination with an alkaline extract of histoplasma capsulatum packaged in glucan particles confers protective immunity in mice. Vaccine (2018) 36(23):3359–67. doi: 10.1016/j.vaccine.2018.04.047 PMC5960637 29729993

[42] Yotsuya S Shikama H Imamura M. Efficacy of the inflammatory cell infiltration inhibitor is-741 on colitis induced by dextran sulfate sodium in the rat. Jpn J Pharmacol (2001) 87(2):151–7. doi: 10.1254/jjp.87.151 11700014

[43] Bolhassani A Talebi S Anvar A. Endogenous and exogenous natural adjuvants for vaccine development. Mini Rev Med Chem (2017) 17(15):1442–56. doi: 10.2174/1389557517666170228115801 28245781

[44] Gu P Xu S Zhou S Liu Z Sun Y Ou N. Optimization of angelica sinensis polysaccharide-loaded poly (Lactic-Co-Glycolicacid) nanoparticles by rsm and its immunological activity in vitro. Int J Biol Macromol (2018) 107(Pt A):222–9. doi: 10.1016/j.ijbiomac.2017.08.176 28867235

[45] Bo R Liu Z Zhang J Gu P Ou N Sun Y. Mechanism of lycium barbarum polysaccharides liposomes on activating murine dendritic cells. Carbohydr Polym (2019) 205:540–9. doi: 10.1016/j.carbpol.2018.10.057 30446138

[46] Lacaille-Dubois MA. Updated insights into the mechanism of action and clinical profile of the immunoadjuvant qs-21: A review. Phytomedicine (2019) 60:152905. doi: 10.1016/j.phymed.2019.152905 31182297 PMC7127804

[47] Ho NI Huis In 't Veld LGM Raaijmakers TK Adema GJ. Adjuvants enhancing cross-presentation by dendritic cells: The key to more effective vaccines? Front Immunol (2018) 9:2874. doi: 10.3389/fimmu.2018.02874 30619259 PMC6300500

[48] Yuan D Yuan Q Cui Q Liu C Zhou Z Zhao H. Vaccine adjuvant ginsenoside Rg1 enhances immune responses against hepatitis b surface antigen in mice. Can J Physiol Pharmacol (2016) 94(6):676–81. doi: 10.1139/cjpp-2015-0528 27095502

